# Trends in childhood obesity and central adiposity between 1998-2001 and 2010-2012 according to household income and urbanity in Korea

**DOI:** 10.1186/s12889-015-2616-2

**Published:** 2016-01-07

**Authors:** Jinwook Bahk, Young-Ho Khang

**Affiliations:** 1 Institute of Health Policy and Management, Seoul National University Medical Research Center, Seoul, South Korea; 2 Department of Health Policy and Management, Seoul National University College of Medicine, 103 Daehak-ro, Jongno-gu, Seoul 110-799 South Korea

**Keywords:** Body mass index, Waist circumference, Obesity, Socioeconomic position, Child, Korea, Trends

## Abstract

**Background:**

This study examined trends in body mass index (BMI), waist circumference (WC), and childhood overweight and obesity prevalence between 1998–2001 and 2010–2012 according to household income and urbanity among nationally representative Korean children and adolescents aged 10-19.

**Methods:**

The repeated cross-sectional data from Korean National Health and Nutrition Examination Surveys in 1998-2001 and 2010-2012 were used. Gender specific trends in age-adjusted means of WC and BMI by household equivalized income and urbanity were compared between years. The age-standardized prevalence of childhood overweight and obesity was calculated using three international criteria (International Obesity Task Force, World Health Organization, US Centers for Disease Control and Prevention) and a Korean national reference standard.

**Results:**

Among boys, overall BMI and overweight prevalence increased between 1998–2001 and 2010–2012, while overall WC decreased. Clear gender differences were found in the relationship of childhood obesity metrics with household income and urbanity and the time trends of those relationships. Positive relationships between these parameters were found for boys while negative relationships appeared for girls. In addition, compared with the childhood obesity prevalence among boys in rural areas, the prevalence among boys in urban areas were slightly lower in 1998–2001 but became greater in 2010–2012.

**Conclusions:**

This study revealed gender difference in the association of childhood obesity with household income and urbanity and its time trends. The long-term gender-specific monitoring of socioeconomic and urban-rural differences in childhood obesity measures is warranted in South Korea.

**Electronic supplementary material:**

The online version of this article (doi:10.1186/s12889-015-2616-2) contains supplementary material, which is available to authorized users.

## Background

Childhood obesity is associated with increased risks of premature mortality and cardiovascular morbidity in adulthood [1, 2]. Encouragingly, recent studies, especially from developed countries, have reported stabilization or declining trends in childhood obesity prevalence [3]. However, these stabilizations or reductions in childhood obesity might be unequally distributed across sub-populations with different socioeconomic positions (SEP) and/or in different geographical regions. Prior studies from Australia and England have shown that individuals with higher SEP showed stabilized or declining trends in childhood obesity, while those with lower SEP showed increasing trends [4, 5]. Major cities in the Netherlands and Switzerland showed stabilized or decreased childhood overweight prevalence over recent decades, whereas other smaller cities and rural areas showed increasing prevalence [6, 7]. Information on time trends in childhood obesity measures according to SEP and geographic regions (e.g., urban vs. rural areas) may indicate priority groups for intervention.

Body mass index (BMI) has been commonly used to define childhood overweight and obesity [8], but waist circumference (WC) is an important index of central adiposity in childhood as well [9]. BMI and WC may have similar abilities in predicting future cardiometabolic risk profiles [10]. However, it is unclear if childhood BMI and WC show the same secular trends. Moreover, to the best of our knowledge, no studies have examined the time trends of both childhood obesity and central adiposity according to SEP and urbanity.

In South Korea (hereafter ‘Korea’), the prevalence of overweight and obesity among children and adolescents has stabilized since the early 2000s [11]. It is unclear whether time trends in childhood overweight and obesity and central adiposity differ according to SEP and urbanity. To answer this question, we explored changes in BMI, WC, and the prevalence of childhood overweight and obesity according to household income and urbanity between 1998–2001 and 2010–2012, using repeated cross-sectional nationally representative samples of 6016 Korean children and adolescents aged 10–19.

## Methods

### Study subjects

Data were derived from the first (1998), second (2001), and fifth (2010–12) waves of the Korean National Health and Nutrition Examination Survey (K-NHANES). The K-NHANES microdata are publicly available through the official website of KNHANES (http://knhanes.cdc.go.kr). We combined the first and second wave data from 1998 and 2001 to obtain a value representative of the situation circa 2000. K-NHANES was a repeated cross-sectional survey on a representative national sample which was based on multi-stage clustered probability samples from Korean households representing the civilian non-institutionalized population [12]. The response rates for K-NHANES were 86.5 % in 1998, 77.3 % in 2001, and 76.5 % in 2010–2012. Additional details of K-NHANES are described elsewhere [12, 13]. Data from 6016 participants (3110 boys and 2906 girls) aged 10–19 (3175 in 1998–2001 and 2841 in 2010–12) were analyzed, after excluding 376 individuals without anthropomorphic data or information on household income and urbanity. The Institutional Review Board of the Seoul National University Hospital, Seoul, Korea, approved this study.

### Household income and urbanity

Information on household income was obtained with questions on monthly and annual income. A question on monthly income was used in 1998 and 2001 while questions on both annual and monthly income were employed in 2010–2012. Household income was adjusted for family size (gross household income divided by the square root of the number of household members) and then classified into 3 categories (low, middle, and high) according to tertile distributions in each year. Residential areas were categorized into urban and rural areas, using the administrative classification of the Ministry of Public Administration and Security in South Korea. The basic administrative unit, *dong* in Korean, from major metropolitan cities and small- and medium-sized cities was considered as urban areas while other basic administrative units, *eup* and *myon* in Korean, were considered as rural areas.

### Anthropometric measurement

The anthropometric measures included height, weight, and WC. Measurements were conducted with the same protocols and anthropometric measurement instruments for all waves of K-NHANES [13]. Body weight was measured to the nearest 0.1 kg on a calibrated balance-beam scale while the participants wore a lightweight gown or underwear. Height was measured in the upright position to the nearest 0.1 cm using a stadiometer. WC was measured to the nearest 0.1 cm with measurement tape placed horizontal to the floor without indenting the skin. BMI was calculated as weight (kg) divided by squared height (m^2^). BMI Z-scores were obtained using the method recommended by the US Centers for Disease Control and Prevention (US CDC) [14].

### Criteria for childhood overweight and obesity

In most of this study, we used two different criteria for childhood overweight and obesity based on BMI measurements: the International Obesity Task Force (IOTF) criteria and the Korea Centers for Disease Control and Prevention (KCDC) criteria. In the additional files (see Additional file 1: Table S1 and Additional file 2: Table S2), we also presented results using the US CDC and World Health Organization (WHO) criteria for childhood overweight and obesity. The IOTF criterion was adapted based on the extrapolation of adult BMI cut-off points for overweight (25 kg/m^2^) and obesity (30 kg/m^2^) from children living in six countries. We used the IOTF cut-offs at age 18, while for the age of 19, we used the same cut-offs as for adults (overweight ≥ 25 kg/m^2^ and obesity ≥ 30 kg/m^2^), because specific BMI cut-offs for the age of 19 do not exist in the IOTF criteria [15]. The KCDC criteria also employed age- and gender-specific BMI values and defined the 85th percentile as the cut-off for “overweight” and the 95th percentile as the cut-off for “obesity” [16]. A prior Korean study employed these four criteria and found similar time trends of childhood obesity prevalence between criteria [11]. In this study, we employed the four criteria and examined any differences by criteria in the relationship of childhood obesity measures with household income and urbanity and its time trends.

### Statistical analysis

Using the R statistical programming language, we created figures for BMI Z-score distributions according to household income and urbanity by survey year and sex using its smoothing function. Using SAS version 9.3 (SAS Institute, Cary, NC, USA), we analyzed gender-specific changes in the age-adjusted least square mean (± standard error) of BMI and WC according to household income and urbanity, using regression analysis (PROC SURVEYREG in SAS) after taking into account sample weights for K-NHANES. To analyze changes in the age-adjusted prevalence (95 % confidence intervals [CI]) of childhood overweight and obesity, we employed the direct standardization method using the 2010 Korean Census population as the standard population. Sample weights were also taken into account in this standardization. In this study, the prevalence of overweight includes the prevalence of obesity. We used logistic regression analyses (PROC SURVEYLOGISTIC in SAS) to estimate *P* values for between-group differences (household income groups and urban-rural areas) and time trends between 1998–2001 and 2010–2012. We also examined changes in the magnitude of prevalence difference (absolute difference) in childhood obesity and overweight by computing the *P* values of the interactions between time period and household income (year*household income) and between time period and urbanity (year*urbanity), using a log-binomial regression with PROC GENMOD in the SAS statistical software [17]. When the binomial model of the changes in prevalence difference failed to converge (i.e., changes in the prevalence difference by urbanity in boys according to the IOTF criteria), the modified Poisson approach was used [17].

## Results

Table 1 shows the distribution of study subjects according to household income and urbanity. The proportion of subjects living in rural areas decreased by about 11 percentage points among both boys and girls between 1998–2001 and 2010–2012 (Table 1).

**Table 1:** Year- and gender-specific numbers (percentage) of study subjects according to household income and urbanity

	1998–2001	2010–2012	Total
Boys	1622 (100.0)	1488 (100.0)	3110 (100.0)
Household income			
Low	531 (32.7)	473 (31.8)	1004 (32.3)
Middle	550 (33.9)	490 (32.9)	1040 (33.4)
High	541 (33.4)	525 (35.3)	1066 (34.3)
Urbanity			
Rural	410 (25.3)	207 (13.9)	617 (19.8)
Urban	1212 (74.7)	1281 (86.1)	2493 (80.2)
Girls	1553 (100.0)	1353 (100.0)	2906 (100.0)
Household income			
Low	518 (33.4)	476 (35.2)	994 (34.2)
Middle	528 (34.0)	457 (33.8)	985 (33.9)
High	507 (32.6)	420 (31.0)	927 (31.9)
Urbanity			
Rural	399 (25.7)	202 (14.9)	601 (20.7)
Urban	1154 (74.3)	1151 (85.1)	2305 (79.3)

### Changes in body mass index Z-scores by household income and urbanity

**Fig. 1:**
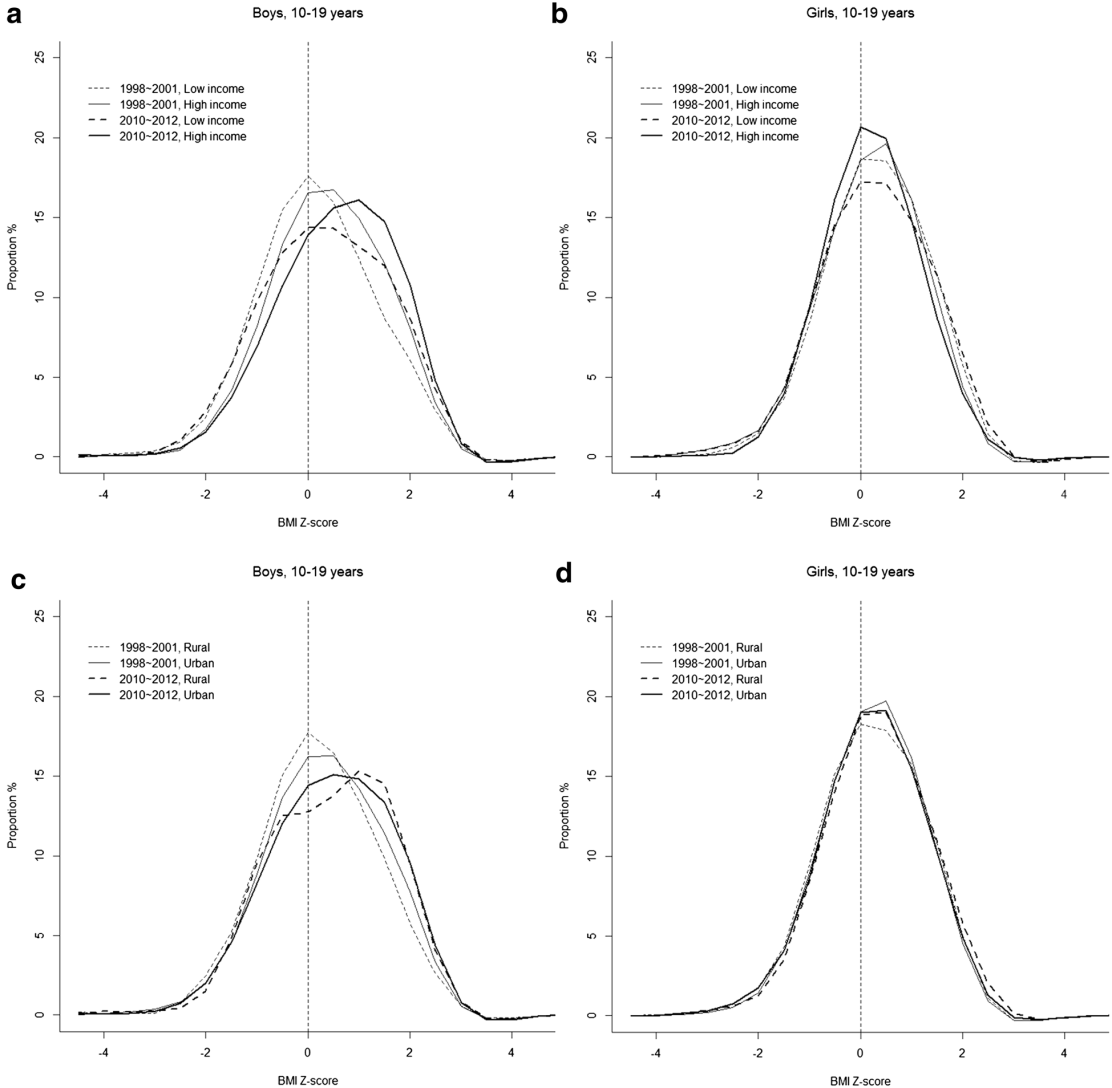
Changes in the distribution of body mass index (BMI) Z-scores among boys and girls aged 10–19, according to household income status (high vs. low) (**a** for boys and **b** for girls) and urbanity (**c** for boys and **d** for girls) between 1998–2001 and 2010–2012

Figure 1 presents changes in the distribution of BMI Z-scores among boys and girls in 1998–2001 and 2010–2012 according to household income status (high vs. low) and urbanity. In 2010–2012, a large part of the BMI Z-score distributions were positive among boys regardless of their household income status and urbanity. Among boys with a low household income, the distribution of BMI Z-scores was in the middle of the graph (0 points in the BMI Z-score) in 1998–2001 but widened and shifted toward the right side in 2010–2012. Among boys with a high household income, the distribution showed no apparent widening but shifted toward the right in 2010–2012. A similar shift toward the right was observed for the distributions of BMI Z-scores according to urbanity among boys. Meanwhile, among girls, no apparent shift toward the right of the distribution in the BMI Z-scores was found according to household income status and urbanity (Fig. 1).

### Trends in body mass index and waist circumferences by household income and urbanity

Table 2 presents trends in the least square mean BMI values among boys and girls between 1998–2001 and 2010–2012, after taking into account age distributions and sample weight. Overall, BMI increased in boys (*P* for trend = 0.0195) but plateaued in girls (*P* for trend = 0.3435). Among boys, the BMI increase between 1998–2001 and 2010–2012 was statistically significant among those with high household income and urban residency (*P* for trend = 0.0175 and 0.0379, respectively), whereas BMI values among girls have stabilized irrespective of SEP and urbanity. Table 2 also shows gender differences in the relationships of BMI with household income and urbanity. Among boys, BMI was positively associated with household income in both 1998–2001 and 2010–2012 (*P* values for trend = 0.0101 and 0.0017, respectively). However, these positive relationships were not found among girls. Rather, a negative relationship was found between household income and BMI in girls, especially in 1998–2001 (*P* value = 0.0215). In addition, boys residing in urban areas showed greater BMI values than boys with rural residences, although the urban-rural difference was only statistically significant in 1998–2001 (*P* value = 0.0087 in 1998–2001 and 0.083 in 2010–2012). Among girls, no statistical difference in BMI according to urbanity was found (*P* value = 0.5636 in 1998–2001 and 0.1752 in 2010–2012) (Table 2). Additional file 3: Table S3 also shows changes in the BMI Z-scores over the study period by household income and urbanity and provides generally similar findings as Table 2 does for BMI.

**Table 2:** Trends in the least square mean (± standard error) values for body mass index and waist circumference according to household income and urbanity

	1998–2001	2010–2012	*P* for trends*	*P* for interaction**
Body mass index				
Boys	20.42 (0.09)	20.76 (0.11)	0.0195	
Household income				
Low	20.09 (0.15)	20.38 (0.20)	0.2431	0.3864
Middle	20.46 (0.15)	20.73 (0.19)	0.2676	
High	20.67 (0.16)	21.28 (0.20)	0.0175	
*P*-value for trend***	0.0101	0.0017		
Urbanity				
Rural	19.96 (0.18)	20.31 (0.27)	0.291	0.9616
Urban	20.52 (0.10)	20.86 (0.13)	0.0379	
*P*-value****	0.0087	0.083		
Girls	20.01 (0.08)	20.14 (0.11)	0.3435	
Household income				
Low	20.34 (0.16)	20.26 (0.19)	0.7782	0.7072
Middle	19.86 (0.13)	20.17 (0.18)	0.1607	
High	19.87 (0.14)	19.94 (0.18)	0.7738	
*P*-value for trend***	0.0215	0.188		
Urbanity				
Rural	20.03 (0.17)	20.47 (0.32)	0.2438	0.3532
Urban	20.00 (0.10)	20.07 (0.11)	0.6463	
*P*-value****	0.5636	0.1752		
Waist circumference (cm)				
Boys	71.59 (0.25)	70.75 (0.30)	0.0308	
Household income				
Low	70.93 (0.43)	69.98 (0.51)	0.1527	0.3942
Middle	71.57 (0.41)	70.37 (0.49)	0.0592	
High	72.20 (0.45)	72.13 (0.56)	0.9133	
*P*-value for trend***	0.039	0.0053		
Urbanity				
Rural	70.32 (0.48)	69.69 (0.73)	0.4722	0.7682
Urban	71.87 (0.28)	70.97 (0.33)	0.0385	
*P*-value****	0.0071	0.1363		
Girls	67.41 (0.22)	66.85 (0.26)	0.0952	
Household income				
Low	68.46 (0.39)	67.09 (0.46)	0.0244	0.1762
Middle	67.18 (0.36)	66.94 (0.41)	0.6587	
High	66.69 (0.36)	66.41 (0.42)	0.62	
*P*-value for trend***	0.0007	0.2872		
Urbanity				
Rural	67.25 (0.42)	67.83 (0.71)	0.4802	0.122
Urban	67.44 (0.25)	66.63 (0.27)	0.0272	
*P*-value****	0.8649	0.0869		

*P-values for time trends between 1998–2001 and 2010–2012

**P-values for the interactions between time period and household income and between time period and urbanity

***P-values for linear trends among household income groups

****P-values for between-group (urban-rural areas) differences

Table 2 also presents trends in the least square mean WC values among boys and girls. In contrast to the BMI trends, WC decreased in boys (*P* for trend = 0.0308) and stabilized in girls (P for trend = 0.0952) between 1998–2001 and 2010–2012. Both boys and girls living in urban areas showed a significantly decreasing WC trend (*P* value for WC trend = 0.0385 in boys and 0.0272 in girls). Girls with low household income also presented a significant decrease in WC (*P* for trends = 0.0244). The results of the analysis of the relationships of WC with household income and urbanity showed clear gender differences. Among boys, WC increased linearly with household income in both time intervals (*P* values for trend = 0.039 in 1998–2001 and 0.0053 in 2010–2012, respectively), whereas the reverse relationship appeared in girls (*P* values for trend = 0.0007 in 1998–2001 and 0.2872 in 2010–2012). A similar gender difference was found for the urban-rural difference in WC. Boys with urban residency showed greater WC than boys with rural residency in 1998–2001 (*P* value = 0.0071), while the WC of girls with rural residency tended to be greater than that of girls with urban residency in 2010–2012 (*P* value = 0.0869) (Table 2).

Although Table 2 and Additional file 3: Table S3 show significant gender differences in the relationships of BMI, WC, and BMI Z-score with household income and urbanity, no apparent statistical time trends in the magnitude of those relationships over the period investigated were found. All P values for interactions of time periods with household income and urbanity were > 0.05.

### Trends in the prevalence of childhood obesity by household income and urbanity

Table 3 shows trends in the age-standardized prevalence (95 % CI) of childhood obesity between 1998–2001 and 2010–2012 using two different criteria (IOTF’s international criteria and KCDC’s local criteria). Based on the IOTF criteria, boys with high household income and urban residency showed significant increases in childhood obesity prevalence between 1998–2001 and 2010–2012 (*P* value for trend = 0.0435 and 0.0205, respectively). Among girls, the overall prevalence of childhood obesity according to the IOTF criteria showed a significantly increasing trend from 1.3 to 2.7 % (*P* for trend = 0.0141) (Table 3).

**Table 3:** Trends in age-standardized prevalence (95 % confidence intervals) of childhood obesity according to household income and urbanity among South Korean boys and girls aged 10–19

	1998–2001	2010–2012	*P* for trends*	*P* for interaction**		1998–2001	2010–2012	*P* for trends*	*P* for interaction**
Boys					Girls				
IOTF criteria	3.4 (2.5–4.3)	4.8 (3.5–6.1)	0.0751		IOTF criteria	1.3 (0.7–1.8)	2.7 (1.6–3.9)	0.0141	
Household income					Household income				
Low	3.2 (1.7–4.7)	4.1 (2.3–5.9)	0.4392	0.4249	Low	2.1 (0.9–3.4)	3.6 (1.5–5.6)	0.211	0.8101
Middle	3.3 (1.9–4.8)	3.7 (1.5–5.8)	0.8356		Middle	0.8 (0.1–1.5)	2.0 (0.2–3.7)	0.2291	
High	3.5 (1.9–5.1)	6.5 (3.8–9.2)	0.0435		High	1.1 (0.1–2.0)	2.2 (0.6–3.8)	0.1393	
*P*-value for trend***	0.9189	0.1771			*P*-value for trend***	0.1519	0.245		
Urbanity					Urbanity				
Rural	3.8 (1.8–5.7)	1.9 (0.2–3.6)	0.171	0.0117	Rural	2.6 (1.0–4.3)	8.1 (2.7–13.5)	0.1346	0.3353
Urban	3.3 (2.3–4.3)	5.4 (3.8–6.9)	0.0205		Urban	1.0 (0.4–1.5)	2.1 (1.1–3.1)	0.0536	
*P*-value****	0.6957	0.0197			*P*-value****	0.0342	0.0387		
KCDC 2007	5.2 (4.0–6.4)	6.9 (5.3–8.5)	0.1427		KCDC 2007	5.9 (4.5–7.2)	7.8 (6.0–9.6)	0.101	
Household income					Household income				
Low	4.3 (2.5–6.0)	6.4 (3.8–8.9)	0.2611	0.6890	Low	8.6 (5.6–11.5)	9.3 (6.3–12.3)	0.7375	0.9588
Middle	4.9 (3.0–6.7)	5.4 (2.9–7.9)	0.8176		Middle	3.7 (2.1–5.4)	6.2 (3.3–9.1)	0.1703	
High	6.2 (3.8–8.6)	8.7 (5.6–11.9)	0.2113		High	5.6 (3.5–7.7)	7.3 (4.3–10.2)	0.3357	
*P*-value for trend***	0.2758	0.2521			*P*-value for trend***	0.1134	0.2516		
Urbanity					Urbanity				
Rural	5.7 (3.3–8.1)	3.9 (1.2–6.7)	0.2371	0.0356	Rural	7.1 (4.5–9.7)	13.5 (7.2–19.8)	0.1686	0.3428
Urban	5.1 (3.8–6.4)	7.4 (5.6–9.2)	0.0526		Urban	5.6 (4.1–7.2)	7.2 (5.4–9.0)	0.2448	
*P*-value****	0.6814	0.0484			*P*-value****	0.4082	0.1444		

*P-values for time trends between 1998–2001 and 2010–2012

**P-values for the interactions between time period and household income and between time period and urbanity

***P-values for linear trends among household income groups

****P-values for between-group (urban-rural areas) differences

Table 3 also presents gender differences in the relationship of childhood obesity with household income and urbanity. A positive relationship between household income and childhood obesity was found in boys while a negative relationship appeared in girls. However, those relationships between household income and childhood obesity did not reach statistical significance. Statistically significant relationships were found for urban-rural differences. While boys in urban areas showed a greater prevalence of childhood obesity than boys in rural areas according to both IOTF and KCDC criteria in 2010–2012 (*P* values = 0.0197 and 0.0484, respectively), girls living in rural areas had a greater prevalence of IOTF criteria-based childhood obesity than girls in urban areas in 1998–2001 and 2010–2012 (*P* values = 0.0342 and 0.0387, respectively) (Table 3).

Table 3 also reveals a widening gap in the prevalence of childhood obesity, especially according to urbanity. Based on the IOTF criteria, the absolute gap of childhood obesity prevalence between urban and rural areas in boys was -0.5 % (3.3 % minus 3.8 %) in 1998–2001 but increased to 3.5 % (5.4 % minus 1.9 %) in 2010–2012 and the interaction of urbanity and time period for the changes in the prevalence difference was statistically significant (*P* for interaction = 0.0117). Based on the KCDC criteria, the same interaction of urbanity and time period in boys also reached statistical significance (*P* for interaction = 0.0356). Meanwhile, the directionality of this increasing gap was the opposite in girls. Based on the IOTF criteria, the absolute gap in the prevalence of childhood obesity between urban and rural areas in girls was -1.6 % (1.0 % minus 2.6 %) in 1998–2001 but −6.0 % (2.1 % minus 8.1 %) in 2010–2012. However, the interaction of urbanity and time period was not statistically significant (*P* value for interaction = 0.3353). Analyses of household income also produced similar results to those of urbanity. Disparities according to household income develop in opposite directions according to gender, but there was no statistically significant interaction between household income and time period (Table 3). In addition, when we conducted additional analyses using the US CDC and WHO criteria, the findings were generally similar to the results based on IOTF and KCDC criteria, but a significant increase in the urban-rural gap of childhood obesity among girls according to WHO criteria was observed (*P* for interaction = 0.0483) (Additional file 1: Table S1).

### Trends in the prevalence of childhood overweight by household income and urbanity

Table 4 presents trends in the age-standardized prevalence (95 % CI) of childhood overweight (including obesity) between 1998–2001 and 2010–2012 using IOTF and KCDC criteria. Among boys, the overall overweight prevalence significantly increased from 20.4 to 25.6 % according to the IOTF criteria between 1998–2001 and 2010–2012 (*P* for trend = 0.0027). Boys in both low and high household income groups and boys with urban residency showed significant increases in overweight prevalence over this period (*P* value for trend = 0.0145, 0.0208, and 0.0061 respectively). Meanwhile, among girls, overweight prevalence stabilized during the same period and no significant trends were noted according to household income and urbanity (Table 4).

**Table 4:** Trends in age-standardized prevalence (95 % confidence intervals) of childhood overweight according to household income and urbanity among South Korean boys and girls aged 10–19

	1998–2001	2010–2012	*P* for trends*	*P* for interaction**		1998–2001	2010–2012	*P* for trends*	*P* for interaction**
Boys					Girls				
IOTF criteria	20.4 (18.3–22.5)	25.6 (22.9–28.3)	0.0027		IOTF criteria	15.0 (13.1–17.0)	16.7 (14.3–19.1)	0.3184	
Household income					Household income				
Low	16.4 (13.0–19.7)	23.7 (19.3–28.1)	0.0145	0.9315	Low	18.7 (14.9–22.6)	19.8 (15.7–23.9)	0.6558	0.9977
Middle	21.7 (18.0–25.3)	24.4 (19.9–28.9)	0.4186		Middle	13.5 (10.4–16.6)	15.8 (11.5–20.1)	0.6519	
High	22.2 (18.5–25.9)	29.4 (24.7–34.1)	0.0208		High	13.3 (10.3–16.2)	13.9 (9.9–17.9)	0.8108	
*P*-value for trend***	0.0263	0.0701			*P*-value for trend***	0.0434	0.0355		
Urbanity					Urbanity				
Rural	15.9 (12.1–19.7)	20.1 (14.5–25.8)	0.2276	0.7877	Rural	14.4 (10.9–17.9)	21.7 (14.6–28.8)	0.1915	0.2495
Urban	21.4 (19.0–23.8)	26.7 (23.8–29.6)	0.0061		Urban	15.1 (12.9–17.3)	16.3 (13.7–18.9)	0.6156	
*P*-value****	0.0186	0.0743			*P*-value****	0.7173	0.3666		
KCDC 2007	15.2 (13.3–17.2)	18.5 (16.1–20.9)	0.0514		KCDC 2007	16.9 (14.9–18.9)	18.2 (15.7–20.8)	0.4672	
Household income					Household income				
Low	13.7 (10.6–16.9)	17.4 (13.4–21.4)	0.2613	0.4813	Low	21.4 (17.5–25.4)	20.9 (16.7–25.1)	0.9034	0.6819
Middle	14.8 (11.6–18.0)	15.6 (11.7–19.5)	0.7925		Middle	15.9 (12.6–19.2)	18.3 (13.6–22.9)	0.6741	
High	16.7 (13.3–20.1)	22.9 (18.5–27.3)	0.0345		High	13.8 (10.7–16.8)	14.9 (10.8–19.0)	0.6374	
*P*-value for trend***	0.2927	0.0687			*P*-value for trend***	0.005	0.0493		
Urbanity					Urbanity				
Rural	12.1 (8.6–15.6)	13.8 (8.7–18.8)	0.6597	0.5495	Rural	17.5 (13.6–21.3)	24.5 (17.3–31.7)	0.301	0.3593
Urban	15.9 (13.7–18.1)	19.4 (16.7–22.0)	0.0571		Urban	16.8 (14.5–19.0)	17.6 (14.9–20.3)	0.7501	
*P*-value****	0.0619	0.0673			*P*-value****	0.7772	0.2463		

*P-values for time trends between 1998–2001 and 2010–2012

**P-values for the interactions between time period and household income and between time period and urbanity

***P-values for linear trends among household income groups

****P-values for between-group (urban-rural areas) differences

As in Tables 3, 4 also shows gender differences in the relationship of childhood obesity with household income and urbanity. Childhood overweight was positively related with household income in boys (*P* value for trend = 0.0263 in 1998–2001 and 0.0701 in 2010–2012), whereas negative associations between household income and overweight were found among girls (*P* value for trend = 0.0434 in 1998–2001 and 0.0355 in 2010–2012). Boys in urban areas showed a greater overweight prevalence than boys in rural areas according to both IOTF and KCDC criteria in 1998–2001 and 2010–2012. Meanwhile, more girls living in rural areas tended to be overweight than girls in urban areas, especially in 2010–2012 (Table 4). However, these relationships between urbanity and childhood overweight were not statistically significant except for boys in 1998–2001. Analyses based on the USCDC and WHO criteria showed the same patterns of difference by gender (Additional file 2: Table S2).

Table 4 presents a tendency for childhood overweight differentials according to urbanity to widen, as seen in Table 3. The absolute gap of overweight prevalence between urban and rural areas in boys increased by 1.1 percentage points between 1998–2001 and 2010–2012 (5.5 % in 1998–2001 and 6.6 % in 2010–2012) based on the IOTF criteria, and increased by 1.8 percentage points (3.8 % in 1998–2001 and 5.6 % in 2010–2012) based on the KCDC criteria. Meanwhile, the absolute gap of childhood overweight prevalence between urban and rural areas in girls tended to increase, but the directionality of this trend was opposite to that in boys. Based on the IOTF criteria, the absolute gap in the prevalence of childhood obesity between urban and rural areas in girls was 0.7 % (15.1 % minus 14.4 %) in 1998–2001 but widened to −5.4 % (16.3 % minus 21.7 %) in 2010–2012. This trend was also observed according to the KCDC criteria (−0.7 % in 1998–2001 and −6.9 % in 2010–2012). However, the interaction of urbanity and time period were not statistically significant in either boys or girls (Table 4).

## Discussion

The results of this study showed gender differences in the relationship of childhood obesity with household income and urbanity. Childhood obesity and overweight tended to be more prevalent in boys with high household income or urban residency and in girls with low household income or rural residency. Similar gender differences were also found for WC and BMI Z-score. These clear gender differences in Korea are interesting because, in recent years, positive relationships between adiposity measures and SEP have become uncommon, particularly in developed countries [18]. A recent study conducted in Poland showed similar gender differences with respect to SEP in relation with WC and BMI [19]. Two explanations are possible. One possible explanation is that body image perception and associated weight control behavior differ according to gender and SEP [20]. For example, a recent Korean study has shown that weight misperception was more prevalent among girls than boys (57.9 % of girls overestimated their weight) and girls with a high SEP were more likely to overestimate their weight status [21]. Meanwhile, weight misperception might have been less influential in explaining weight control behaviors in Korean boys unlike in Korean girls. For example, Korean girls were more likely to engage in weight control behaviors for weight loss than Korean boys [22]., Korean girls were also more likely to try various dieting practices, even including unhealthy methods, than Korean boys [23]. In addition, an international paper showed that, among Korean university students, the prevalences of perceived overweight and trying to lose weight were three times greater in women than in men [24]. Of total 22 countries, the prevalence of ‘trying to lose weight’ was highest in Korean female university students [24]. Levels of calorie intake and physical activity have been shown to differ according to SEP [25]. Another possible explanation is that these gender differences might have originated from earlier childhood. In Korea, huge urban-rural inequalities have existed. The relative disadvantage in economic development, health behaviors, health outcomes, and health care resources have been observed in rural area [26]. Adolescents with low household income or rural residency might have been exposed to disadvantaged socioeconomic environments from their early childhood. A recent study showed that developmental trajectories of BMI are established between ages 1 and 4 years, no directional change occurs between 4 and 10 years, and then the trajectories persist between 10 and 18 years of age [27]. Moreover, the impact of the early childhood socioeconomic environment on later obesity and associated etiologically relevant periods may differ by gender [28, 29]. Prior studies from France and the Netherlands showed strong associations of early life social disadvantages with adulthood obesity in women but not in men [28, 29]. Residual associations between early childhood SEP and obesity measures were evident in women not in men [30]. In this regard, studies examining the relationship between SEP and obesity in earlier childhood (i.e., ages 1 and 10 years) are warranted to explain gender difference patterns in Korean children and adolescents.

Results of this study showed different time trends in childhood overweight and obesity by household income and urbanity. Prior studies from England, France, and Sweden demonstrating increasing socioeconomic inequalities in childhood obesity and overweight have indicated differences in the time trends of childhood obesity according to individual SEP [4, 31, 32]. Other studies from the Netherlands and Switzerland have shown different time trends of childhood overweight by the size of cities [6, 7]. These studies showed stabilized or decreasing trends in childhood obesity among high SEP groups and major cities but increasing trends among low SEP groups and smaller cities [4, 6, 7, 31, 32]. These results from other countries seem to show opposite results among boys regarding widening disparities according to SEP and urbanity. It is difficult to fully understand the mechanisms of the differently widening disparities in childhood obesity inequalities among Korean boys, but we suppose that these findings in Korean boys may have resulted from the abovementioned gender differences in body perception and early childhood factors. Among South Korean girls aged 10–19, early childhood socioeconomic disadvantages, which would be a strong predictor of low household income and rural residency, might be associated with adolescent obesity, as prior studies from other countries suggested [28, 29]. However, this would not be the case for South Korean boys. For example, gradual secular increases in the energy intake from fat and associated westernized dietary patterns were observed from 1998 to 2005 among Korean adolescents aged 10–19 years [33]. Meanwhile, the westernized dietary pattern and the percentage of energy from fat were positively associated with abdominal obesity and overweight only in Korean boys [33]. In addition, rural boys were more likely to prefer thin body shapes than urban boys, whereas urban girls reported a stronger preference to a skinny body than rural girls [34]. In this regard, the overall increases in obesity and overweight in Korean adolescents might be partly attributable to high calories intake, but the gender differences in socioeconomic obesity inequalities might be explained by different effects of body image preferences and early childhood factors among boys and girls.

One interesting result of this study is that the time trends for BMI and WC between 1998–2001 and 2010–2012 were opposite, especially among boys. Boys’ overall BMI and overweight prevalence increased between 1998–2001 and 2010–2012, while overall WC decreased. One possible explanation is that the increased ‘body mass’ was not adipose mass, but rather lean mass [35]. Considering that overall body weight and height have also increased between 1998–2001 and 2010–2012 among boys (see Additional file 3: Table S3), these results might indicate that Korean boys became larger without increases in central adiposity.

One of the strengths of this study is that it was based on nationally representative data containing consistent anthropometric measurements of height, weight, and WC, which were determined from examinations using the same protocols and instruments from samples of 10– to 19-year-old Korean children and adolescents. Moreover, to the best of our knowledge, this is one of the first studies to explore socioeconomic differentials in obesity trends using both BMI-based measures and WC. Furthermore, we used both international (IOTF, US CDC, and WHO) and local (KCDC) criteria for the prevalence of childhood overweight (including obesity) and obesity. By doing so, we were able to compare trends in total body mass distribution and abdominal adiposity at a given time.

However, this study also has some limitations. Since the sample size for rural areas, especially in 2010–2012, was small, the results for rural areas showed relatively large standard errors and CIs. We think it is likely that it was for this reason that the time trends between 1998–2001 and 2010–2012 in rural areas generally were not significant in most analyses. To confirm this, further studies with large sample sizes should be conducted in the future. In addition, another limitation of this study is that we only used household income as a SEP measure. Several studies suggested that stronger associations were noted between SEP and childhood obesity according to parental education level or occupational social class than according to household income [36, 37]. Studies using other childhood SEP measures, such as maternal education or parental occupational position, are warranted in the future.

## Conclusions

We examined trends in childhood overweight, obesity, BMI, and WC in nationally representative samples of South Korean children and adolescents aged 10–19 between 1998–2001 and 2010–2012, according to household equivalized income and urbanity. The results indicate that clear gender differences existed among Korean children and adolescents regarding the socioeconomic patterning of childhood obesity. Opposite time trends in socioeconomic disparities in childhood obesity according to gender were also observed. Gender differences in body perception and the role of early childhood factors might be a possible explanation for some of these trends. The prevalence of obesity among Korean children and adolescents was not as high as the prevalence found in other developed countries [38]. However, considering the widening absolute gap in childhood overweight and obesity prevalence between urban and rural areas and between high and low SEP groups, the long-term monitoring of socioeconomic patterning in childhood overweight and obesity along with effective policies for narrowing socioeconomic disparities in childhood overweight and obesity are needed.

## Additional files

Additional file 1: Table S1. Trends in age-standardized prevalence (95 % confidence intervals) of childhood obesity according to household income and urbanity among South Korean boys and girls aged 10–19. (DOCX 27 kb)

Additional file 2: Table S2. Trends in age-standardized prevalence (95 % confidence intervals) of childhood overweight according to household income and urbanity among South Korean boys and girls aged 10–19. (DOCX 27 kb)

Additional file 3: Table S3. Trends in least square mean (± standard error) values for weight, height, and body mass index (BMI) Z score according to household income and urbanity. (DOCX 17 kb)

## Abbreviations

BMI: Body mass index
CI: confidence intervals
IOTF: International Obesity Task Force
KCDC: Korea Centers for Disease Control and Prevention
K-NHANES: Korean National Health and Nutrition Examination Survey
SEP: socioeconomic positions
US CDC: US Centers for Disease Control and Prevention
WC: waist circumference
WHO: World Health Organization

## References

[1] Park MH, Falconer C, Viner RM, Kinra S (2012). The impact of childhood obesity on morbidity and mortality in adulthood: a systematic review. Obes Rev.

[2] Reilly JJ, Kelly J (2011). Long-term impact of overweight and obesity in childhood and adolescence on morbidity and premature mortality in adulthood: systematic review. Int J Obes.

[3] Olds T, Maher C, Zumin S, Péneau S, Lioret S, Castetbon K (2011). Evidence that the prevalence of childhood overweight is plateauing: data from nine countries. Int J Pediatr Obes.

[4] Stamatakis E, Wardle J, Cole TJ (2010). Childhood obesity and overweight prevalence trends in England: evidence for growing socioeconomic disparities. Int J Obes.

[5] Rokholm B, Baker JL, Sørensen TI (2010). The levelling off of the obesity epidemic since the year 1999-a review of evidence and perspectives. Obes Rev.

[6] Murer SB, Saarsalu S, Zimmermann MB, Aeberli I (2014). Pediatric adiposity stabilized in Switzerland between 1999 and 2012. Eur J Nutr.

[7] Schonbeck Y, Talma H, van Dommelen P, Bakker B, Buitendijk SE, HiraSing RA (2011). Increase in prevalence of overweight in Dutch children and adolescents: a comparison of nationwide growth studies in 1980, 1997 and 2009. PLoS One.

[8] Lobstein T, Baur L, Uauy R, IASO International Obesity TaskForce. Obesity in children and young people: a crisis in public health. Obes Rev. 2004;5 Suppl 1:4–104. 10.1111/j.1467-789X.2004.00133.x 15096099

[9] Taylor RW, Jones IE, Williams SM, Goulding A (2000). Evaluation of waist circumference, waist-to-hip ratio, and the conicity index as screening tools for high trunk fatmass, as measured by dual-energy X-ray absorptiometry, in children aged 3–19 y. Am J Clin Nutr.

[10] Reilly JJ, Kelly J, Wilson DC (2010). Accuracy of simple clinical and epidemiological definitions of childhood obesity: systematic review and evidence appraisal. Obes Rev.

[11] Khang YH, Park MJ (2011). Trends in obesity among Korean children using four different criteria. Int J Pediatr Obes.

[12] Kweon S, Kim Y, Jang MJ, Kim Y, Kim K, Choi S (2014). Data resource profile: the Korea National Health and Nutrition Examination Survey (KNHANES). Int J Epidemiol.

[13] KCDC. Korean National Health and Nutrition Examination Survey. Korea Centers for Disease Control and Prevention., Chungcheongbuk-do, Korea. 2014. http://knhanes.cdc.go.kr/. Accessed 24 Jun 2014.

[14] Kuczmarski RJ, Ogden CL, Guo SS, Grummer-Strawn LM, Flegal KM, Mei Z, et al. 2000 CDC Growth Charts for the United States: methods and development. Vital and health statistics Series 11, Data from the national health survey. 2002(246):1-190. 12043359

[15] Cole TJ, Bellizzi MC, Flegal KM, Dietz WH. Establishing a standard definition for child overweight and obesity worldwide: international survey. BMJ. 2000;320(7244):1240–3. 10.1136/bmj.320.7244.1240 PMC27365 10797032

[16] KCDC, The Korean Pediatric Society, The Committee for the Development of Growth Standard for Korean Children and Adolescents. 2007 Korean children and adolescents growth standard (commentary for the development of 2007 growth chart). Government report online [in Korean]. Seoul: Korea Centers for Disease Control and Prevention; 2007.

[17] Spiegelman D, Hertzmark E. Easy SAS calculations for risk or prevalence ratios and differences. Am J Epidemiol. 2005;162(3):199–200. 10.1093/aje/kwi188 15987728

[18] Shrewsbury V, Wardle J. Socioeconomic status and adiposity in childhood: a systematic review of cross-sectional studies 1990-2005. Obesity. 2008;16(2):275–84. 10.1038/oby.2007.35 18239633

[19] Gurzkowska B, Kulaga Z, Litwin M, Grajda A, Swiader A, Kulaga K (2014). The relationship between selected socioeconomic factors and basic anthropometric parameters of school-aged children and adolescents in Poland. Eur J Pediatr.

[20] Lim HJ, Wang YF (2013). Body weight misperception patterns and their association with health-related factors among adolescents in South Korea. Obesity.

[21] Lim HJ, Wang YF (2013). Body weight misperception patterns and their association with health-related factors among adolescents in South Korea. Obesity.

[22] Ha Y, Choi E, Seo Y, Kim TG (2013). Relationships among subjective social status, weight perception, weight control behaviors, and weight status in adolescents: findings from the 2009 Korea Youth Risk Behaviors Web-Based Survey. J School Health.

[23] Lim H, Lee HJ, Park S, Kim CI, Joh HK, Oh SW (2014). Weight misperception and its association with dieting methods and eating behaviors in South Korean adolescents. Nutr Res Pract.

[24] Wardle J, Haase AM, Steptoe A (2005). Body image and weight control in young adults: international comparisons in university students from 22 countries. Int J Obes.

[25] Frederick CB, Snellman K, Putnam RD (2014). Increasing socioeconomic disparities in adolescent obesity. Proc Natl Acad Sci U S A.

[26] Kim CY, Kim MH, Lee TJ, Son JI (2015). Inequality in health: a Korean perspective.

[27] Ziyab AH, Karmaus W, Kurukulaaratchy RJ, Zhang H, Arshad SH (2014). Developmental trajectories of Body Mass Index from infancy to 18 years of age: prenatal determinants and health consequences. J Epidemiol Community Health.

[28] Giskes K, van Lenthe FJ, Turrell G, Kamphuis CBM, Brug J, Mackenbach JP (2008). Socioeconomic position at different stages of the life course and its influence on body weight and weight gain in adulthood: a longitudinal study with 13-year follow-up. Obesity.

[29] Khlat M, Jusot F, Ville I (2009). Social origins, early hardship and obesity: a strong association in women, but not in men?. Soc Sci Med.

[30] Chapman BP, Fiscella K, Duberstein P, Coletta M, Kawachi I (2009). Can the influence of childhood socioeconomic status on men’s and women’s adult body mass be explained by adult socioeconomic status or personality? Findings from a National sample. Health Psychol.

[31] Lioret S, Touvier M, Dubuisson C, Dufour A, Calamassi-Tran G, Lafay L (2009). Trends in child overweight rates and energy intake in France from 1999 to 2007: relationships with socioeconomic status. Obesity.

[32] Sundblom E, Petzold M, Rasmussen F, Callmer E, Lissner L (2008). Childhood overweight and obesity prevalences levelling off in Stockholm but socioeconomic differences persist. Int J Obes.

[33] Song Y, Park MJ, Paik HY, Joung H (2010). Secular trends in dietary patterns and obesity-related risk factors in Korean adolescents aged 10–19 years. Int J Obes.

[34] Kim YK, Shin WS (2008). A comparison study on perception of body image and dietary habits of high school students between urban and rural areas. Korean J Commun Nutr.

[35] Park H, Park K, Kim MH, Kim GS, Chung S (2011). Gender differences in relationship between fat-free mass index and fat mass index among Korean children using body composition chart. Yonsei Med J.

[36] El-Sayed AM, Scarborough P, Galea S (2012). Socioeconomic inequalities in childhood obesity in the United Kingdom: a systematic review of the literature. Obes Facts.

[37] Bammann K, Gwozdz W, Lanfer A, Barba G, De Henauw S, Eiben G (2013). Socioeconomic factors and childhood overweight in Europe: results from the multi-centre IDEFICS study. Pediatr Obes.

[38] Ng M, Fleming T, Robinson M, Thomson B, Graetz N, Margono C (2014). Global, regional, and national prevalence of overweight and obesity in children and adults during 1980–2013: a systematic analysis for the Global Burden of Disease Study 2013. Lancet.

