# Supplementary material for: Trends in childhood obesity and central adiposity between 1998-2001 and 2010-2012 according to household income and urbanity in Korea
Source: BMC Public Health. 2016 Jan 7;16:18. doi: 10.1186/s12889-015-2616-2 (PMC4705619; doi:10.1186/s12889-015-2616-2)
Supplement: Supplementary file 2 — Trends in age-standardized prevalence (95 % confidence intervals) of childhood overweight according to household income and urbanity among South Korean boys and girls aged 10–19. (DOCX 27 kb) [file 12889_2015_2616_MOESM2_ESM.docx]

Additional file 2: Table S3. Trends in age-standardized prevalence (95% confidence intervals) of childhood overweight according to household income and urbanity among South Korean boys and girls aged 10-19.

|  | 1998-2001 | 2010-2012 | P for trends^a^ | P for interaction^b^ |  |  | 1998-2001 | 2010-2012 | P for trends^a^ | P for interaction^b^ |
| --- | --- | --- | --- | --- | --- | --- | --- | --- | --- | --- |
| **Boys** |  |  |  |  |  | **Girls** |  |  |  |  |
| **U.S. CDC** | 20.1 (18.0-22.2) | 24.7 (22.1-27.3) | 0.0053 |  |  | **U.S. CDC** | 13.2 (11.4-15.0) | 14.8 (12.5-17.1) | 0.3446 |  |
| Household income | |  |  |  |  | Household income | |  |  |  |
| Low | 15.8 (12.6-19.1) | 23.6 (19.3-27.9) | 0.0064 | 0.4095 |  | Low | 16.7 (13.0-20.3) | 17.6 (13.7-21.6) | 0.7114 | 0.6112 |
| Middle | 21.9 (18.3-25.6) | 24.2 (19.7-28.7) | 0.5477 |  |  | Middle | 10.8 (8.2-13.5) | 14.7 (10.6-18.8) | 0.3292 |  |
| High | 21.6 (17.9-25.2) | 26.6 (22.4-30.8) | 0.0597 |  |  | High | 12.4 (9.5-15.3) | 11.4 (7.9-14.9) | 0.7367 |  |
| P-value for trend^c^ | 0.0308 | 0.2441 |  |  |  | P-value for trend^c^ | 0.1125 | 0.027 |  |  |
| Urbanity |  |  |  |  |  | Urbanity |  |  |  |  |
| Rural | 16.0 (12.3-19.7) | 19.9 (14.3-25.4) | 0.2738 | 0.6190 |  | Rural | 13.9 (10.5-17.4) | 19.0 (12.2-25.9) | 0.5136 | 0.6737 |
| Urban | 21.0 (18.6-23.4) | 25.7 (22.9-28.5) | 0.0104 |  |  | Urban | 13.0 (11.0-15.1) | 14.6 (12.2-17.0) | 0.4581 |  |
| P-value^d^ | 0.0295 | 0.0964 |  |  |  | P-value^d^ | 0.6749 | 0.5533 |  |  |
| **WHO** | 25.8 (23.6-28.1) | 29.8 (27.1-32.5) | 0.0323 |  |  | **WHO** | 17.6 (15.6-19.6) | 19.1 (16.6-21.6) | 0.3849 |  |
| Household income | |  |  |  |  | Household income | |  |  |  |
| Low | 20.3 (16.8-23.9) | 26.4 (21.9-30.9) | 0.0718 | 0.8188 |  | Low | 21.5 (17.5-25.5) | 23.5 (19.2-27.7) | 0.4354 | 0.9196 |
| Middle | 27.3 (23.4-31.2) | 29.7 (25.0-34.5) | 0.4502 |  |  | Middle | 16.5 (13.2-19.8) | 17.6 (13.2-22.0) | 0.9949 |  |
| High | 28.7 (24.8-32.6) | 34.2 (29.6-38.9) | 0.0949 |  |  | High | 15.1 (12.0-18.2) | 14.8 (10.8-18.9) | 0.9216 |  |
| P-value for trend^c^ | 0.0032 | 0.0146 |  |  |  | P-value for trend^c^ | 0.0241 | 0.0043 |  |  |
| Urbanity |  |  |  |  |  | Urbanity |  |  |  |  |
| Rural | 20.6 (16.5-24.6) | 27.3 (20.8-33.8) | 0.0695 | 0.4374 |  | Rural | 18.7 (14.8-22.7) | 23.5 (16.2-30.7) | 0.5928 | 0.6857 |
| Urban | 26.9 (24.3-29.5) | 30.2 (27.2-33.2) | 0.1208 |  |  | Urban | 17.3 (15.0-19.6) | 18.8 (16.2-21.5) | 0.4768 |  |
| P-value^d^ | 0.0123 | 0.5633 |  |  |  | P-value^d^ | 0.5505 | 0.5395 |  |  |

^a^ P-values for time trends between 1998-2001 and 2010-2012; ^b^ P-values for the interactions between time period and household income and between time period and urbanity; ^c^ P-values for linear trends among household income groups; ^d^ P values for between-group (urban-rural areas) differences.

Note: the U.S. Centers for Disease Control and Prevention (U.S. CDC) growth charts are derived from five national surveys conducted between 1963 and 1994 in the US. We defined overweight as BMI >85th percentile and obesity as BMI ≥95th percentile (Kuczmarski et al., 2002). The World Health Organization (WHO) criteria were generated from data collected from the WHO Multicentre Growth Reference Study in six countries ([WHO Multicentre Growth Reference Study Group 2006](#_ENREF_32)). In this criteria, BMI values of +1 SD are equivalent to the overweight cut-offs used for adults (≥25.0 kg/m²) and +2 SD corresponds to the cut-off for obesity (≥30.0 kg/m²) (de Onis et al., 2007).
