# Supplementary material for: Trends in childhood obesity and central adiposity between 1998-2001 and 2010-2012 according to household income and urbanity in Korea
Source: BMC Public Health. 2016 Jan 7;16:18. doi: 10.1186/s12889-015-2616-2 (PMC4705619; doi:10.1186/s12889-015-2616-2)
Supplement: Supplementary file 3 — Trends in least square mean (± standard error) values for weight, height, and body mass index (BMI) Z score according to household income and urbanity. (DOCX 17 kb) [file 12889_2015_2616_MOESM3_ESM.docx]

Additional file 3: Table S1. Trends in least square mean (± standard error) values for weight, height, and body mass index (BMI) Z score according to household income and urbanity.

|  | 1998-2001 | 2010-2012 | P for trends | P for interaction |
| --- | --- | --- | --- | --- |
| **Body weight** |  |  |  |  |
| Boys | 54.88 (0.30) | 56.31 (0.39) | 0.0039 |  |
| Household income |  |  |  |  |
| Low | 53.75 (0.53) | 55.17 (0.67) | 0.1007 | 0.6444 |
| Middle | 54.89 (0.52) | 56.27 (0.65) | 0.103 |  |
| High | 55.86 (0.52) | 57.82 (0.66) | 0.0197 |  |
| P-value for trend | 0.0045 | 0.0048 |  |  |
| Urbanity |  |  |  |  |
| Rural | 53.08 (0.61) | 55.06 (1.01) | 0.0964 | 0.5878 |
| Urban | 55.27 (0.34) | 56.58 (0.42) | 0.0163 |  |
| P-value | 0.0024 | 0.2011 |  |  |
| Girls | 48.91 (0.24) | 49.73 (0.31) | 0.0359 |  |
| Household income |  |  |  |  |
| Low | 49.71 (0.45) | 49.85 (0.56) | 0.8438 | 0.4403 |
| Middle | 48.64 (0.38) | 49.93 (0.49) | 0.0394 |  |
| High | 48.48 (0.42) | 49.39 (0.51) | 0.1633 |  |
| P-value for trend | 0.0249 | 0.432 |  |  |
| Urbanity |  |  |  |  |
| Rural | 48.64 (0.47) | 50.37 (0.87) | 0.0799 | 0.3016 |
| Urban | 48.95 (0.27) | 49.59 (0.33) | 0.1352 |  |
| P-value | 0.8089 | 0.2931 |  |  |
| **Height** |  |  |  |  |
| Boys | 162.63 (0.22) | 163.33 (0.26) | 0.038 |  |
| Household income |  |  |  |  |
| Low | 162.28 (0.36) | 163.13 (0.44) | 0.1365 | 0.6993 |
| Middle | 162.44 (0.39) | 163.25 (0.49) | 0.1901 |  |
| High | 163.13 (0.38) | 163.68 (0.42) | 0.324 |  |
| P-value for trend | 0.1064 | 0.3634 |  |  |
| Urbanity |  |  |  |  |
| Rural | 161.70 (0.40) | 162.91 (0.77) | 0.1614 | 0.4865 |
| Urban | 162.83 (0.25) | 163.43 (0.27) | 0.106 |  |
| P-value | 0.0218 | 0.6064 |  |  |
| Girls | 155.72 (0.19) | 156.46 (0.21) | 0.0079 |  |
| Household income |  |  |  |  |
| Low | 155.70 (0.31) | 156.11 (0.38) | 0.3957 | 0.2391 |
| Middle | 155.86 (0.33) | 156.63 (0.33) | 0.0951 |  |
| High | 155.62 (0.33) | 156.84 (0.36) | 0.0099 |  |
| P-value for trend | 0.6827 | 0.2322 |  |  |
| Urbanity |  |  |  |  |
| Rural | 155.23 (0.34) | 156.24 (0.55) | 0.1132 | 0.6452 |
| Urban | 155.83 (0.21) | 156.51 (0.23) | 0.0264 |  |
| P-value | 0.1358 | 0.6735 |  |  |
| **BMI Z score** |  |  |  |  |
| Boys | 0.08 (0.03) | 0.16 (0.04) | 0.0714 |  |
| Household income |  |  |  |  |
| Low | -0.03 (0.05) | 0.01 (0.06) | 0.6271 | 0.2278 |
| Middle | 0.09 (0.05) | 0.18 (0.06) | 0.24 |  |
| High | 0.16 (0.05) | 0.34 (0.06) | 0.0226 |  |
| P-value for trend | 0.0049 | 0.0002 |  |  |
| Urbanity |  |  |  |  |
| Rural | -0.08 (0.06) | 0.02 (0.10) | 0.3898 | 0.8446 |
| Urban | 0.11 (0.03) | 0.19 (0.04) | 0.1181 |  |
| P-value | 0.0056 | 0.1397 |  |  |
| Girls | 0.00 (0.03) | 0.02 (0.03) | 0.6963 |  |
| Household income |  |  |  |  |
| Low | 0.09 (0.05) | 0.03 (0.05) | 0.3828 | 0.3942 |
| Middle | -0.03 (0.04) | 0.05 (0.05) | 0.2195 |  |
| High | -0.04 (0.05) | -0.02 (0.05) | 0.7942 |  |
| P-value for trend | 0.0403 | 0.5116 |  |  |
| Urbanity |  |  |  |  |
| Rural | -0.01 (0.05) | 0.08 (0.08) | 0.3365 | 0.3736 |
| Urban | 0.00 (0.03) | 0.00 (0.03) | 0.9979 |  |
| P-value | 0.9806 | 0.2777 |  |  |

^a^ P-values for time trends between 1998-2001 and 2010-2012; ^b^ P-values of the interactions between time period and household income (year*household income) and between time period and urbanity (year*urbanity); ^c^ P-values for linear trends among household income groups; ^d^ P-values for between-group (urban-rural areas) differences.
